# fNIRS-based functional connectivity estimation using semi-metric analysis to study decision making by nursing students and registered nurses

**DOI:** 10.1038/s41598-020-79053-z

**Published:** 2020-12-16

**Authors:** Jie Sheng Chong, Yee Ling Chan, Esther G. M. Ebenezer, Hoi Yen Chen, Masashi Kiguchi, Cheng-Kai Lu, Tong Boon Tang

**Affiliations:** 1 Centre for Intelligent Signal and Imaging Research, Institute of Health and Analytics, Universiti Teknologi PETRONAS, 32610 Bandar Seri Iskandar, Malaysia; 2 Medicine Based Department, Royal College of Medicine Perak, Universiti Kuala Lumpur, 30450 Ipoh, Malaysia; 3 Nursing Programme, Royal College of Medicine Perak, Universiti Kuala Lumpur, 30450 Ipoh, Malaysia; 4 Research & Development Group, Hitachi Ltd., Tokyo, 185-8601 Japan

**Keywords:** Problem solving, Biomedical engineering, Imaging and sensing

## Abstract

This study aims to investigate the generalizability of the semi-metric analysis of the functional connectivity (FC) for functional near-infrared spectroscopy (fNIRS) by applying it to detect the dichotomy in differential FC under affective and neutral emotional states in nursing students and registered nurses during decision making. The proposed method employs wavelet transform coherence to construct FC networks and explores semi-metric analysis to extract network redundancy features, which has not been considered in conventional fNIRS-based FC analyses. The trials of the proposed method were performed on 19 nursing students and 19 registered nurses via a decision-making task under different emotional states induced by affective and neutral emotional stimuli. The cognitive activities were recorded using fNIRS, and the emotional stimuli were adopted from the International Affective Digitized Sound System (IADS). The induction of emotional effects was validated by heart rate variability (HRV) analysis. The experimental results by the proposed method showed significant difference (FDR-adjusted *p* = 0*.*004) in the nursing students’ cognitive FC network under the two different emotional conditions, and the semi-metric percentage (*SMP*) of the right prefrontal cortex (PFC) was found to be significantly higher than the left PFC (FDR-adjusted *p* = 0*.*036). The benchmark method (a typical weighted graph theory analysis) gave no significant results. In essence, the results support that the semi-metric analysis can be generalized and extended to fNIRS-based functional connectivity estimation.

## Introduction

Graph theory has been widely employed in neuroimaging studies such as functional magnetic resonance imaging (fMRI), electroencephalographic (EEG) and functional near-infrared spectroscopy (fNIRS) to better understand the functional connectivity (FC) under various neurological conditions^1–4^. According to the graph theory, a brain network may be described as a graph consisting of nodes, and the connections between the nodes are known as edges^5^. A collection of nodes in a brain network forms a brain region while the strength or synchronicity of the connectivity is usually represented by the weight of the edges. The number of edges connected to a node is referred to as a degree. To quantitatively evaluate the information transmission ability in a network, the shortest path length plays a crucial role in defining network efficiency at both local and global levels^3^.

**Figure 1:**
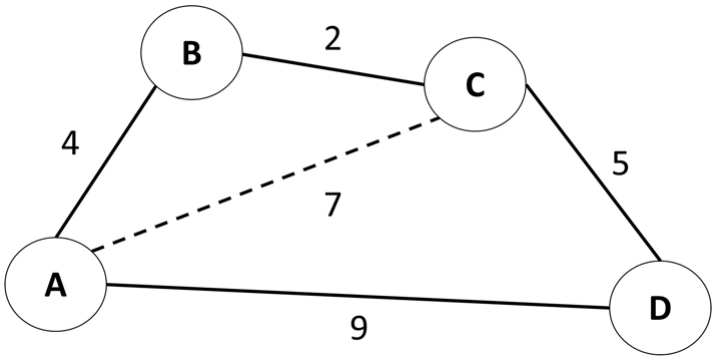
An example of a semi-metric network with weighted path. The dashed line represents the direct path from A to C with a weight of 7. However, there exists a shorter circuitous path from A to C via B with a sum of weight of 6. This is called an indirect path.

In conventional graph theory analysis, the shortest path length is always defined as the minimum sum of the distance between two nodes^6^, providing the most preferable route for information to be passed from one node to another. In real-world networks, the shortest path is not always the direct distance because the distance from one node to another via a circuitous (indirect) path may be less than the length of the direct path^7^. This phenomenon violates the transitive property and forms a semi-metric network^8^, as shown in Fig. 1. Previous studies on fMRI^7,9^ have shown improvement in graphical network processing performance in information sharing by employing a semi-metric network via indirect paths. For instance, semi-metric networks have succeeded in discriminating changes in the human FC in different neurological conditions based on the percentage of the network semi-metricity^8,10,11^. However, its reproducibility and generalizability to other neuroimaging modalities such as fNIRS are unclear.

In semi-metric analysis, a network graph consists of two main components: semi-metric edges and metric backbones. Both these components have different shortest path properties. When a preferential (shortest) route is found via a circuitous path, this indirect path between the nodes is called a semi-metric edge. These semi-metric edges depict a higher level of information sharing among multiple brain regions along the circuitous paths, in contrast to the information exchange by metric backbone edges^10^. This network redundancy feature has not been considered in conventional graph theory-based FC analysis. Such characterization by semi-metric analysis is crucial for describing the hyperconnectivity of the FC. Excessive hyperconnectivity, for instance, has been identified as one of the main symptoms of neurodevelopmental disorder (autism) and post-traumatic stress disorder (emotional processing)^12,13^.

This study proposes the application of fNIRS to measure neuronal activity in the prefrontal cortex (PFC). The fNIRS data was found to closely correlate to that of the fMRI^14^. Importantly, fNIRS devices are generally less expensive, provide higher portability and better balance between spatial and temporal resolution, and also provide higher resistance to motion artifacts^15^. Few studies have been reported using fNIRS to assess functional connectivity during the resting state and cognitive task^2,16,17^. These studies utilized semi-automatic methods for graph filtering, which involved manual tuning of the threshold to select edges above a certain weight value. Such methods may cause inconsistency in the interpretation of FC networks.

In this study, we propose to explore a semi-matrix for fNIRS-based FC analysis for the first time. We hypothesize that (i) the semi-metric analysis applied in fMRI FC estimation could be generalized and extended to fNIRS, and (ii) the semi-metric analysis may provide a more effective technique to evaluate fNIRS-based FC than the conventional graph theory approach. To illustrate, we implemented the proposed method to study the differential FC under affective and neutral emotional states in nursing students and registered nurses.

Studies have shown that nursing is one of the most stressful jobs^18^, and the drop-out rate among nursing students is very high (the average drop-out rate was 35–37% for Italy in 2011, 21.1% for Netherlands in 2013, and 20% for UK in 2015)^19^. The occupational stress results in reduced productivity among nurses (i.e., distracted decision-making ability), threatening patient care. To combat mental stress, registered nurses were found to cope with stress effects by exercising emotion regulation^20^. This study aims to develop a means to detect the dichotomy in differential FC under affective and neutral emotional states in nursing students and registered nurses as the first step to identifying neural markers among nursing students who may be more susceptible to stress coping issues.

## Results

This experiment was conducted in two sessions for each subject (under affective and neutral emotional states), in a counterbalanced manner. Our statistical analysis showed that the FC, HRV, and behavioral performance results were not affected (*p* > 0*.*05) by session order (affective-neutral session versus neutral-affective session).

### HRV analysis

**Figure 2:**
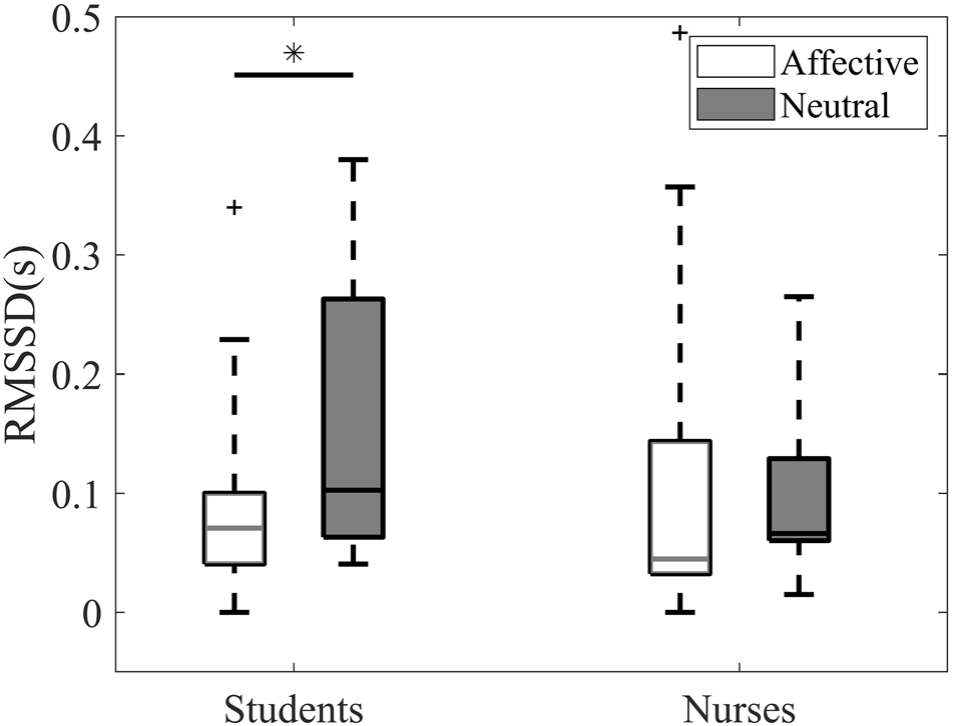
The result of HRV analysis based on *RMSSD*. * indicates FDR-adjusted *p* < 0.05.

Using two-way mixed analysis of variance (ANOVA), the experimental results of root mean square of successive differences (*RMSSD*) showed a two-way interaction [*F*(1,36) = 4.148, *p* = 0.049, ${\eta }_{p}^{2}$ = 0.103] between group type and emotional state. In the subsequent simple effect analysis, the result revealed a significant emotional effect [*F*(1,18) = 8.117, *p* = 0.011, ${\eta }_{p}^{2}$ = 0.311]. The pairwise comparison with false discovery rate (FDR) adjustment (in Fig. 2) showed that nursing students possessed significantly lower *RMSSD* (FDR-adjusted *p* = 0.044, *t*(18) =  − 2.834, Cohen’s *d* = 0.654) in affective state than that in neutral emotional state. On the other hand, no significant difference was observed in *RMSSD* among registered nurses (*p* > 0.05).

### Semi-metric analysis

Based on the two-way mixed ANOVA in global level semi-metric analysis, a significant two-way interaction between group type and emotional state was observed [*F*(1,36) = 5.063, *p* = 0.031,  ${\eta }_{p}^{2}$ = 0.123]. Followed by the simple main effect analysis, significant emotional state effect was only observed in the students [*F*(1,18) = 15.212, *p* = 0.001,  ${\eta }_{p}^{2}$ = 0.458]. From the pairwise comparison with FDR correction (in Table 1), the affective state resulted in significantly higher global *s*emi-metric percentage (*SMP*) (FDR-adjusted *p* = 0.004, *t*(18) = 3.922, Cohen’s *d* = 0.895) than the neutral emotional state among the students. None of the comparison was significantly different in the case of nurses (*p* > 0.05).

**Table 1:** Pairwise comparison between parameters in semi-metric analysis and graph theory analysis at global level.

Comparisons		*df*	*SMP*	*E*_*global*_	^*E*^*local*	*CC*	*λ*
Students	Affective vs. neutral	18	0.004; 3.922; 0.895*	0.760; 0.900; 0.206	0.766; 0.894; 0.205	0.564; 0.895; 0.205	0.590; 1.079; 0.250
Nurses	Affective vs. neutral	18	0.911; 0.113; 0.026	0.760; 1.158; 0.275	0.766; 1.135; 0.275	0.564; 1.136; 0.250	0.590; 1.102; 0.252
Affective	Nurses vs. students	36	0.494; 1.178; 0.393	0.874; 0.159; 0.053	0.899; 0.129; 0.043	0.898; 0.129; 0.043	0.912;0.112; 0.037
Neutral	Nurses vs. students	36	0.548; − 0.831; 0.277	0.847; 0.385; 0.128	0.899; 0.356; 0.119	0.898; 0.356; 0.119	0.912; 0.204; 0.068

The table are shown in (FDR-adjusted *p*-values; *t*-values; Cohen’s *d*). *df* indicates degrees of freedom. * represents FDR-adjusted *p* < 0*.*05.

A three-way mixed ANOVA was carried out to study the interaction between the group type, emotional state, and brain region. The regional semi-metric analysis result indicated a significant three-way interaction effect [*F*(1,36) = 8.278, *p* = 0.007,  ${\eta }_{p}^{2}$ = 0.187]. Based on a follow-up evaluation (by splitting groups into students and nurses), the simple two-way interaction was found to be significant in the students [*F*(1,18) = 20.889, *p* < 0.001, ${\eta }_{p}^{2}$ =  0.537], but not in the nurses [*F*(1,18) = 0.106, *p* = 0.749, ${\eta }_{p}^{2}$ =  0.006]. Result of a follow-up analysis further showed that the students had a significant simple main effect of task [*F*(1,18) = 13.454, *p* = 0.002, ${\eta }_{p}^{2}$ =  0.428] in the right PFC, but not in the left PFC [*F*(1,18) = 0.016, *p* = 0.902, ${\eta }_{p}^{2}$ =  0.001]. The effect of region was also found to be significantly different in the affective state [*F*(1,18) = 9.740, *p* = 0.006, ${\eta }_{p}^{2}$ =  0.351], but not in the neutral emotional state [*F*(1,18) = 1.072, *p* = 0.314, ${\eta }_{p}^{2}$ =  0.056]. Lastly, the multiple pairwise comparison was conducted according to the significant simple main effects. With the FDR correction, Table 2 showed that the *SMP* in the right PFC was significantly greater (FDR-adjusted *p* = 0.036, *t*(18) = 3.113, Cohen’s *d* = 0.716) than that in the left PFC among the students in the affective state. Moreover, in the right PFC, the *SMP* was found to be significantly higher in the affective state than that in the neutral emotional state among the students (FDR-adjusted *p* = 0.024, *t*(18) = 3.298, Cohen’s *d* = 0.841). Meanwhile, none of the comparisons were significantly different in the case of the nurses (*p* > 0.05).

**Table 2:** Pairwise comparison of FC indices in regional semi-metric analysis and graph theory analysis.

Comparisons			*df*	*SMP*	*E*_*nodal*_	*CC*	$\lambda$
Nurses	Affective	Right vs. left PFC	18	0.604; 0.923; 0.212	0.907; 0.316; 0.066	0.928; 0.293; 0.057	0.961; 0.196; 0.033
	Neutral	Right vs. left PFC	18	0.555; 0.919; 0.316	0.907; − 0.326; 0.076	0.928; − 0.332; 0.076	0.948; − 0.382; 0.076
Students	Affective	Right vs. left PFC	18	0.036; 3.113; 0.716*	0.876; 1.069; 0.258	0.858; 1.029; 0.229	0.814; 0.875; 0.204
	Neutral	Right vs. left PFC	18	0.605; 1.036; 0.238	0.876; 0.949; 0.229	0.858; 0.982; 0.367	0.814; 1.114; 0.268
Nurses	Right PFC	Affective vs. neutral	18	0.806; 0.430; 0.099	0.876; 1.565; 0.344	0.858; 1.544; 0.194	0.814; 1.516; 0.344
	Left PFC	Affective vs. neutral	18	0.605; 0.847; 0.194	0.876; 0.868; 0.194	0.858; 0.868; 0.115	0.814; 0.902; 0.212
Students	Right PFC	Affective vs. neutral	18	0.024; 3.298; 0.841*	0.907; 0.555; 0.153	0.928; 0.459; 0.176	0.948; 0.466; 0.098
	Left PFC	Affective vs. neutral	18	0.928; 0.125; 0.029	0.876; 0.793; 0.191	0.858; 0.809; 0.151	0.814; 0.997; 0.229
Affective	Right PFC	Nurses vs. students	36	0.928; − 0.092; 0.031	0.907; − 0.473; 0.158	0.928; − 0.440; 0.147	0.948; − 0.374; 0.125
	Left PFC	Nurses vs. students	36	0.605; 0.765; 0.255	0.995; 0.007; 0.002	0.982; 0.022; 0.007	0.961; 0.050; 0.017
Neutral	Right PFC	Nurses vs. students	36	0.400; 1.734; 0.578	0.876; − 0.798; 0.266	0.858; − 0.819; 0.273	0.814; − 0.840; 0.280
	Left PFC	Nurses vs. students	36	0.605; 0.845; 0.232	0.876; − 0.085; 0.028	0.982; − 0.054; 0.018	0.961; 0.132; 0.044

The values in table are displayed in (FDR-adjusted *p*-values; *t*-values; Cohen’s *d*). *df* represents degrees of freedom. * indicates FDR-adjusted *p* < 0.05.

### Weighted graph theory analysis

Based on the two-way mixed ANOVA conducted separately on the clustering coefficient (*CC*), characteristic path length (*λ*), global efficiency (*E*_*global*_), and local efficiency (*E*_*local*_), we found no significant (*p* > 0.05) results at the global level analysis. At the regional level, three-way mixed ANOVA conducted on the nodal efficiency (*E*_*nodal*_), *CC,* and *λ* revealed that none of the interaction effects (*p* > 0.05) were found to be significant among the three factors (group type, emotional state, and brain region). From the pairwise comparison, as depicted in Table 2, no significant result was observed (*p* > 0.05).

### Behavioral data

From the two-way mixed ANOVA of the behavioral performance parameters, we found no significant results in the number of correctly solved questions, accuracy, and response time. The pairwise comparison results also did not demonstrate any significant differences between the two factors (emotional state and group type).

### Correlation between HRV and FC indices

Based on the Pearson’s correlation analysis, the relationship between HRV and FC indices was assessed. From the results in Table 3, with the adjustment of FDR correction, we identified a significant negative moderate correlation (*r* = − 0.459, FDR-adjusted *p* = 0.020) between changes in the global *SMP* and changes in the *RMSSD*. However, neither of the weighted graph theory indices significantly correlated with the *RMSSD* score.

**Table 3:** Results of correlation between all FC parameters and *RMSSD*.

Parameters	Correlation	FDR-adjusted *p*-values
*SMP*	− 0.459	0.020*
*Eglobal*	− 0.284	0.098
*Elocal*	− 0.278	0.098
*CC*	− 0.277	0.098
*λ*	− 0.275	0.098

*Represents FDR-adjusted *p* < 0.05.

## Discussion

This study introduces a computation of FC (semi-metric analysis), which could be a more effective technique to assess fNIRS-based FC changes due to the affective and neutral emotional states. Firstly, group types were identified in terms of HRV and FC semi-metricity. The significant reduction in the *RMSSD* indicated a clear decrease in parasympathetic activity among the nursing students when in the affective state; on the other hand, the *RMSSD* of the registered nurses did not show any significant change despite changes in emotional states. In terms of behavioral performance, the nurses, exhibiting no significant changes in HRV and FC indices, had no significant difference in the number of correctly solved questions, accuracy, and response time. Likewise, the behavioral performance indices of the students were observed to be insignificant. Proceeding to the FC analyses, the comparison of semi-metric analysis and graph theory analysis in detecting emotional effects was evaluated based on two approaches, including ANOVA and correlation analysis with *HRV*. The conventional weighted graph theory analysis showed no significant results for both nursing students and registered nurse groups. On the other hand, by splitting into individual groups, the semi-metric analysis was able to distinguish significant changes of semi-metricity, especially at the right PFC among the students due to emotional effects.

According to previous studies^21,22^, the significant reduction in HRV among nursing students might be explained by the adaptive physiological responses under the elicitation of external emotional stimuli. In contrast, the non-significant change in ANS activity among the registered nurses, as indicated by HRV values, might reflect that the nurses have developed their own coping strategy and the affective stimulus did not affect decision making (i.e., the task). This validated the induction of emotional states as a reference for FC analyses. Moving on to FC analyses, earlier fMRI studies determined that the increment in *SMP* reflected a higher level of hyperconnectivity and dispersal of FC, which included other brain regions^10^. The presence of hyperconnectivity in PFC areas has been further linked by previous studies to the processing of emotions such as anxiety and stress^23,24^. The apparent changes in brain semi-metricity in the right PFC regions among students might imply the increase in information sharing between the right PFC and other brain regions due to emotional effects. As discussed in several studies^13,25^, lateral PFC areas are involved in the cognitive control of emotion. Our ANOVA results showed an agreement with previous studies^4,13,26^ where the right lateralized asymmetry of FC was expected as the students were exposed to affective stimuli.

Moreover, the study^27^ demonstrated that the non-significant emotional effect on task performance among students was due to the compensatory effort of the subjects. According to the attentional control theory^28^, students tended to maintain their behavioral performance in the affective state by increasing the executive function of PFC, which involved the right lateral PFC (BA 9/46), in agreement with our findings in the regional semi-metric analysis. This explained the emotional effects that were found to be significant in the change of brain topology, but not significant in behavioral performance among students. It also revealed that subjects in the affective state, indicated by a significant drop in the HRV *RMSSD*, possessed a significant reduction in the *SMP*. The linear correlation analysis strengthened the reliability of the semi-metric analysis by detecting a significant moderate negative association between the *SMP* and the *RMSSD*, as shown in Table 3. Therefore, it can be concluded that the affected cognition due to emotion may be detected from changes in the *SMP*.

This study has some limitations. Firstly, the structural description of the semi-metric network remains unclear. In the present study, the semi-metric network was constructed based on the existence of indirect paths, but it is still a challenge to specify all involved paths along all indirect connections. Assessing the differences between the two emotional states may help identify the brain regions involved in emotional cognition. Secondly, the subject groups are significantly different (*p* < 0.001) in age. The age effect could possibly confound with the FC results of group comparisons. Thus, further work would include the structural study of the semi-metric network and the correction of age effect.

## Methods

**Figure 3:**
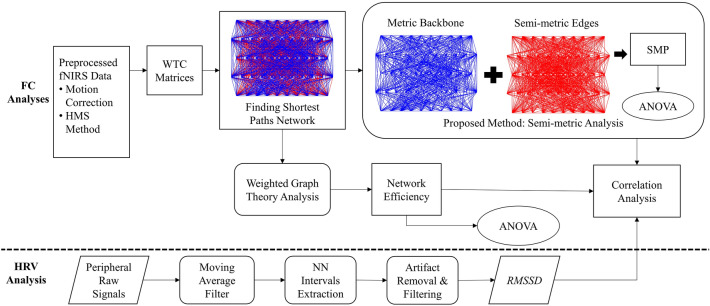
Summary flow chart for FC analyses and HRV analysis. Using the weighted network theory, the derived shortest path was directly quantified into network efficiency. In semi-metric metric analysis, the semi-metric component was extracted to be quantified as *SMP*.

In this section, we present the overall functional connectivity analysis framework, which includes a novel FC analysis using semi-metric and benchmark FC analysis based on weighted graph theory, as illustrated in Fig. 3. The framework utilizes heart rate variability (HRV) to validate the success of inducing the affective state, and statistical tests such as ANOVA and correlation analysis with HRV analysis to compare the effectiveness of both the FC estimation methods.

### Proposed FC estimation method

#### Data preprocessing

First, the motion artifact was eliminated from the optical density (OD) of fNIRS signals using wavelet-based motion correction based on the *hmrMotionCorrectWavelet* function in HOMER2^29^. The OD signal was decomposed into Gaussian distributed wavelet coefficients. Wavelet coefficients exceeding 1.5 times the interquartile range were eliminated as motion artifacts. By converting the corrected OD to ∆*HbO* and ∆*HbR*, we applied a low-pass filter with 1.0 Hz to remove high-frequency noise components. Subsequently, we extracted the functional neuronal component by separating the systemic physiological component (i.e., cerebral blood circulation) from the fNIRS signal based on the hemodynamic modality separation (HMS) method^30^.

#### Functional connectivity matrix

FC refers to the temporal correlation of the interacting cerebral region signals during the cognitive task^31^. In this study, we employed wavelet transform coherence (WTC) to construct brain FC matrices by using MATLAB Wavelet Coherence Toolbox^32^. WTC provided an advanced computation to Pearson’s correlation to measure the time-varying correlation between two signals in the frequency domain. It is suitable to assess non-stationary changes between fNIRS signals, especially the task-associated changes, and it has been widely used to investigate brain FC in fNIRS studies^33,34^.

Based on the separation of the functional signal in the HMS method using the linear relationship assumption between ∆*HbO* and ∆*HbR*, we could expect the same FC matrices for both signal types. To verify our assumption in selecting signal types, we applied the same FC analyses and eventually observed the same results for both functional ∆*HbO* and ∆*HbR* signals. Therefore, we only selected functional ∆*HbO* signals, which are more sensitive to task-related changes, as the backbone of measurement^35^. The analyses of functional ∆*HbR* signals are shown in Supplementary Table 1 to Table 6. The functional ∆*HbO* time series signals were initially decomposed into wavelet coefficients in the time–frequency domain using wavelet transform^36^. Subsequently, we computed the pairwise correlation between all channels’ wavelet coefficients to construct time–frequency dimensional network correlation matrices. The frequency band of interest lies between 0.01 Hz and 0.2 Hz^37^. Within this range of frequency of interest, we extracted and averaged the 60 s task-relevant correlations to construct 32 × 32 network matrices. The channels represent the nodes, whereas the averaged correlation values denote weighted and undirected network edges. The constructed weighted network matrices were then submitted for semi-metric analysis and typical weighted graph theory analysis, as illustrated in Fig. 3.

#### Semi-metric analysis

From the weighted and undirected graphs, we converted the correlation matrix to a distance graph by using a distance conversion function^38,39^ as per Eq. (1):

1 $${l}_{ij}=\frac{1}{{x}_{ij}}-1$$

where *l*_*i j*_ denotes the distance from node *i* to *j* and *x*_*i j*_ is the correlation weight between nodes *i* and *j*, given that the positions of the two different nodes are *i* to *j*.

Subsequently, we labeled the semi-metric edges if *l*_*i j*_ was less than the summation of paths via other nodes between nodes *i* and *j*, for instance: *l*_*ac*_ < *l*_*ab*_ + *l*_*bc*_, given that there are nodes *a*, *b,* and *c*. As described in the pseudocode in Algorithm 1, we initiated the detection of semi-metric edges by finding the shortest paths, *l*^′^, based on the shortest path algorithm (i.e., Johnson’s Algorithm^40^). Next, we computed the ratio of semi-metricity, *s*_*i j*_^10^:

2 $${s}_{ij}=\frac{{l}_{ij}}{{l}_{ij}^{^{\prime}}}$$

*s*_*i j*_ greater than 1 represented semi-metric edges whereas *s*_*i j*_ equivalent to 1 denoted metric backbone edges.
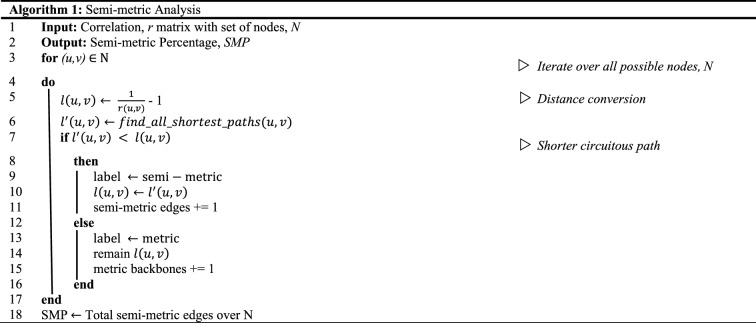


#### Semi-metric properties in FC networks

**Figure 4:**
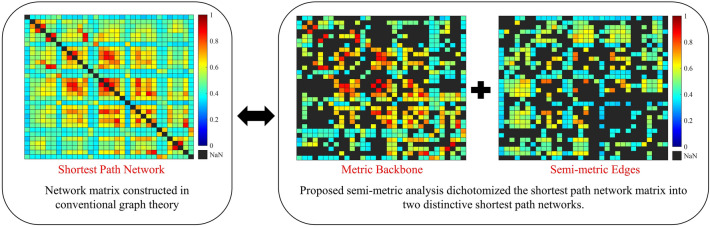
FC maps showing the network matrices involved in conventional graph theory and semi-metric analysis. Further interpretation is conducted in semi-metric analysis where the information about sharing feature in a shortest path matrix is considered.

**Figure 5:**
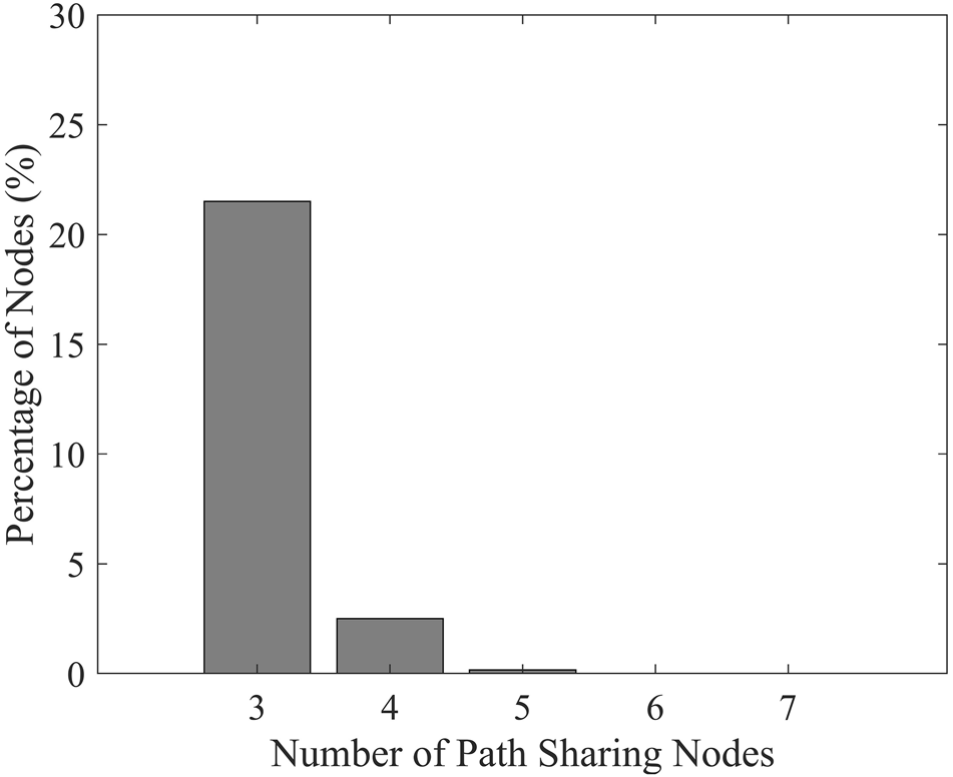
The total number of shared paths used to construct the shortest paths among all subjects.

Finding the shortest paths in FC analysis may utilize two or more nodes to allow direct flow or sharing of information, respectively. Conventional graph theory, which quantifies an FC matrix based on its shortest paths, does not consider path sharing in the shortest paths. The application of semi-metric analysis categorized the shortest paths as either direct paths or sharing paths, as constructed in Fig. 4. A map of semi-metric edges shows information about sharing paths when the number of nodes involved, is greater than two. In detail, Fig. 5 shows that more than 20% of all the shortest paths were constructed by utilizing more than two-node paths, confirming a strong presence of path sharing in the shortest paths.

#### Performance metric

To characterize the semi-metric behavior of the brain network, we calculated the *SMP* based on the semi-metric ratio^10^:

3 $$SMP=\frac{\sum_{i,j}{s}_{ij}>1}{E}$$

where *E* is the total number of connections in the original network. Ultimately, *SMP* values were analyzed statistically at the global and regional levels based on the regions of interest (ROI).

### Weighted graph theory analysis

Acting as a test bench for semi-metric analysis, we performed a typical weighted graph theory approach^2,41^ to explore the reliability of semi-metric analysis in differentiating emotional states. The weighted graph theory analysis was conducted using the Brain Connectivity Toolbox (BCT)^5^. From the unthresholded and weighted functional network, we performed a global analysis by computing the network topological parameters including the *CC*, *λ*, *E*_*global*_, and *E*_*local*_.

When the other nodes around a node of interest form at least a triangular connection, the measure of the cliquishness is defined as *CC*^42^ in Eq. (4):

4 $$CC=\frac{1}{N}\sum_{i}\frac{2m}{{k}_{i}({k}_{i}-1)}$$

From the FC matrices, we computed *λ* to quantify the integration of the potential information flow based on the average shortest path length as per Eq. (5):

5 $$\lambda =\frac{1}{N}\sum_{i\in G}\frac{\sum_{j\in G,j\ne i}{d}_{ij}}{N-1}$$

*E*_*global*_ was described as the inverse of the harmonic mean of the shortest path length within the whole network^43,44^. It quantified the ability of global and concurrent exchange of information between connected edges as derived in Eq. (6):

6 $${E}_{global}=\frac{1}{N(N-1)}\sum_{j\ne i\in G}\frac{1}{{d}_{ij}}$$

The *E*_*local*_ of a network *G* is defined as the mean local efficiency of each node^41,43^ as shown in Eq. (7) It not only characterizes the capability of information flow across node *i* to its nearest neighbor nodes but also reflects the tolerance of neighboring nodes when there is a defect in node *i*.

7 $${E}_{local}=\frac{1}{N}\sum_{i\in G}{E}_{global}({G}_{i})$$

We further decomposed the PFC networks into regional subgraphs based on the ROI. To quantify the information propagation ability across regions, we evaluated the regional *CC*, *λ,* and *E*_*nodal*_ of all nodes within the ROI using Eq. (8)^43^:

8 $${E}_{nodal}=\frac{1}{N-1}\sum_{j\in G}\frac{1}{{d}_{ij}}$$

From the equations above, *i* = 1, 2, 3, *N*; *j* $\ne$ *i* refers to the region relative to node *i*; *m* is the number of neighboring edges; *d*_*i j*_ denotes the weighted shortest path length between nodes *i* and *j*; *N* refers to the total number of nodes in the network, *G*, which consists of all the nodes.

### Validation experiment

#### Subjects

In total, 39 right-handed, healthy nursing subjects, consisting of 19 nurses with actual working experience (Edinburgh Handedness Inventory^45^ scale = 86.18 ± 15.53, age = 30.44 ± 3.20 years old, working experience = 8.32 ± 3.04 years) and 20 students with only internship experience (Edinburgh Handedness Inventory scale = 93.13 ± 12.48, age = 20.68 ± 0.82 years old, internship experience = 2.70 ± 0.41 years) participated in this study. Prior to the experiment, all subjects had to complete a screening questionnaire which included demographic information such as physical health, mental condition and family history of disease. Subjects with known history of any psychiatric or neurological disorders were excluded. The participants were prohibited from consuming alcohol and caffeine, smoking, and exercising for at least 3 h before the experiment. One nursing student who did not fulfill the requirements was excluded. Using G*Power 3^46^, a sensitivity power analysis was carried out to evaluate the sample size based on the repeated measure ANOVA (within-between interaction), given the following conditions: (1) significant level = 0.05, (2) power of 1 − *β* = 0.80, (3) 2 groups, and (4) 2 measurements. The generated minimal detectable effect reported a critical effect size *f*(U) = 0.480 (or ${\eta }_{p}^{2}$ =  0.102). This study was approved by the ethics committee of Universiti Kuala Lumpur Royal College of Medicine Perak (UniKL RCMP) (approval number: UniKLRCMP/MREC/2018/018). All the subjects provided informed consent, and the experiment was carried out in accordance with the Declaration of Helsinki guidelines and regulations.

#### Measurement

**Figure 6:**
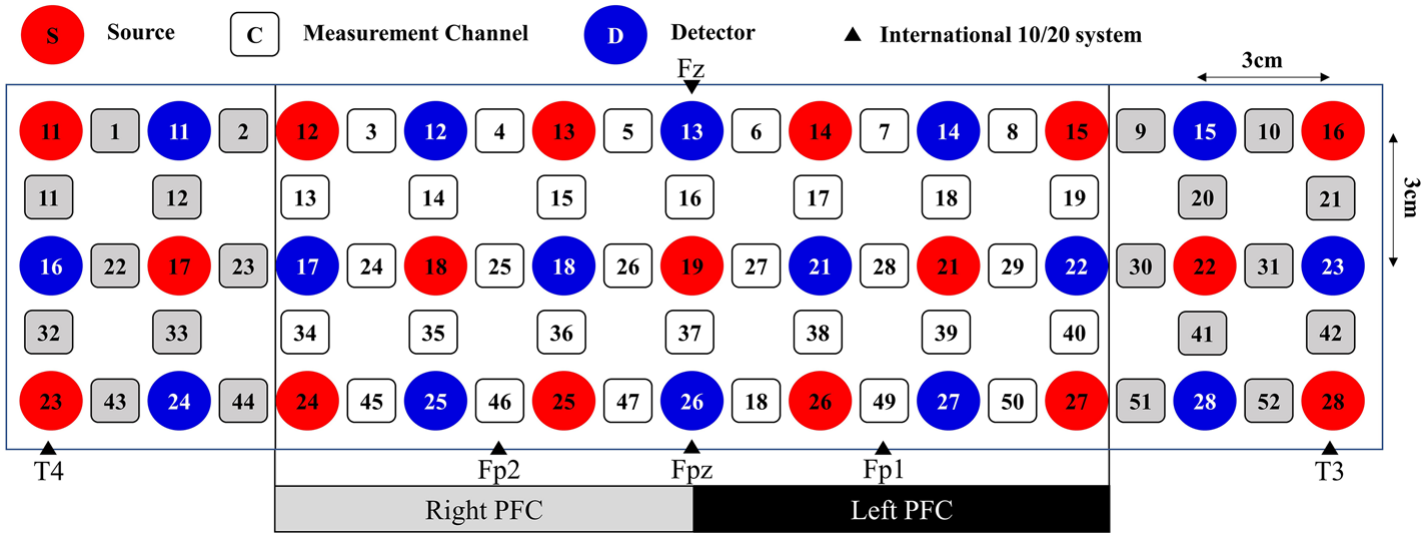
Probes were setup on subjects’ forehead and scalp based on the international 10/20 system.

Brain activity in the PFC was measured using a dual-wavelength (695 nm and 830 nm) multichannel OT-R40 fNIRS continuous wave system (Hitachi Medical Corporation, Japan), with a sampling rate of 10 Hz. A 52-channel 3 × 11 optodes layout (17 sources and 16 detectors) with a source-detector distance of 3 cm was deployed based on the international 10/20 system^47^ along the T4-Fpz-T3 positions. By using distinctive absorption coefficients of different chromophores and the modified Beer-Lambert Law^48^, we calculated the change in the concentration of oxygenated hemoglobin (∆*HbO*) and deoxygenated hemoglobin (∆*HbR*) based on the changes in light intensity of the dual-wavelength light. We estimated the channel localization according to the Montreal Neurological Institute (MNI) coordination, determining the Brodmann area (BA) for each channel^35,47^. Here, we identified PFC regions based on the 32 channels as labeled in Fig. 6 and subsequently divided the regions into the left and right PFC as our ROI. Measurements from channels 16 and 37 were excluded when we compared the two hemispheres.

A Nellcor DS-100A ear clip sensor was placed on the left ear of the subjects. The earclip sensor was connected to the AFE4490SPO2EVM Evaluation Board (Texas Instruments Inc., Dallas, Texas) to collect photoplethysmographic (PPG) signals at a sampling rate of 200 Hz simultaneously with the fNIRS measurement. The purpose of measuring PPG signals was to perform heart rate variability (HRV) analysis. Compared with electrocardiogram (ECG), PPG offers higher simplicity, minimum subject discomfort, and lower cost. Previous studies have reached a consensus that PPG is an alternative to ECG in estimating HRV^49,50^. The evaluation of emotional states based on PPG has also been implemented in a recent study^51^.

#### Affective and neutral emotional stimuli

**Figure 7:**
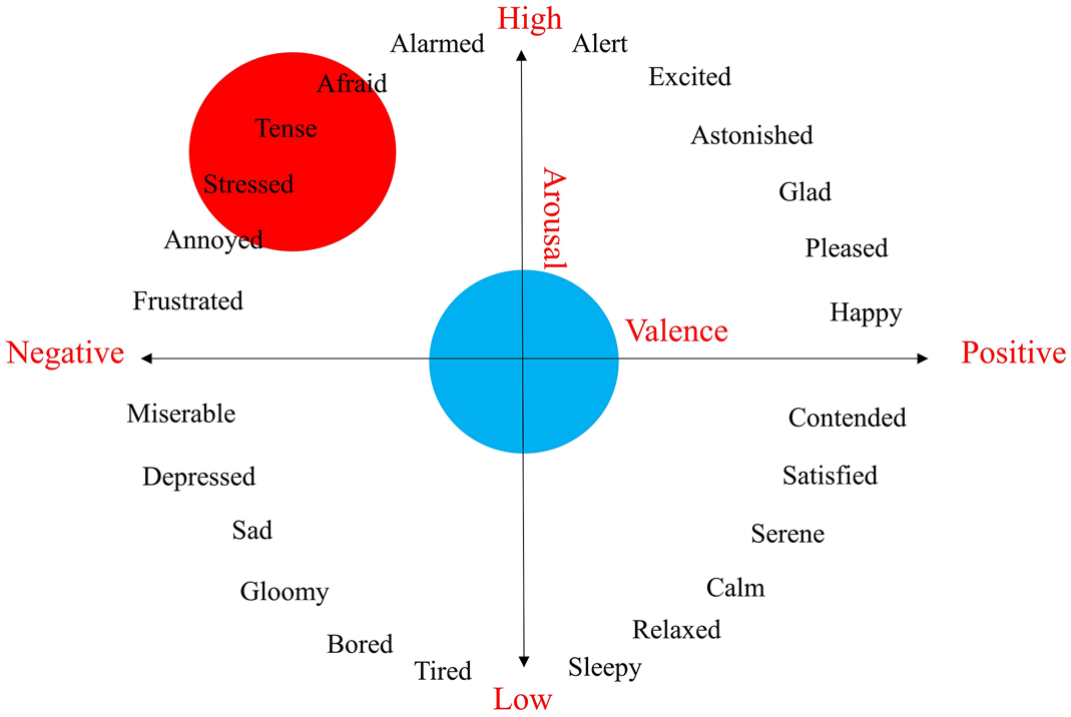
Emotional circumplex model based on the 9-point Self-Assessment Manikin (SAM) ratings classifies various emotional adjectives. The red-shaded and blue-shaded regions indicate the regions of retrieving affective and neutral stimuli respectively.

Two sets of the auditory emotional stimuli were retrieved to induce different emotional states from the International Affective Digitized Sounds system (IADS)^52^. The first set consisting of ten affective sound clips was referred to as the "case" set while the other set of ten neutral sound clips was labelled as the "control" set. The classes of stimuli were based on the emotional circumplex model, as shown in Fig. 7^53^. Descriptively, the emotional circumplex model comprises two independent neurophysiological dimensions, known as valence and arousal ratings. These ratings are scaled according to the Self-Assessment Manikin (SAM) 9-point ratings^54^. The IADS provides a standardized database of emotional stimuli based on two-dimensional ratings. We defined affective stimulus as sound clips audible in hospital with negative valence (rating of 2.147 ± 0.473 out of 9) and high arousal (rating of 7.388 ± 0.494 out of 9) including an ambulance siren, the crying of a baby, and human screams, whereas the neutral stimulus comprised sound clips with neutral valence (rating of 5.197 ± 0.720 out of 9) and medium arousal (rating of 4.560 ± 0.380 out of 9), such as the sounds of typewriting, clock ticking, and raindrops. The stimulus was played in the background throughout the entire task to induce different emotional states (one session, one emotional state).

#### Task

**Figure 8:**
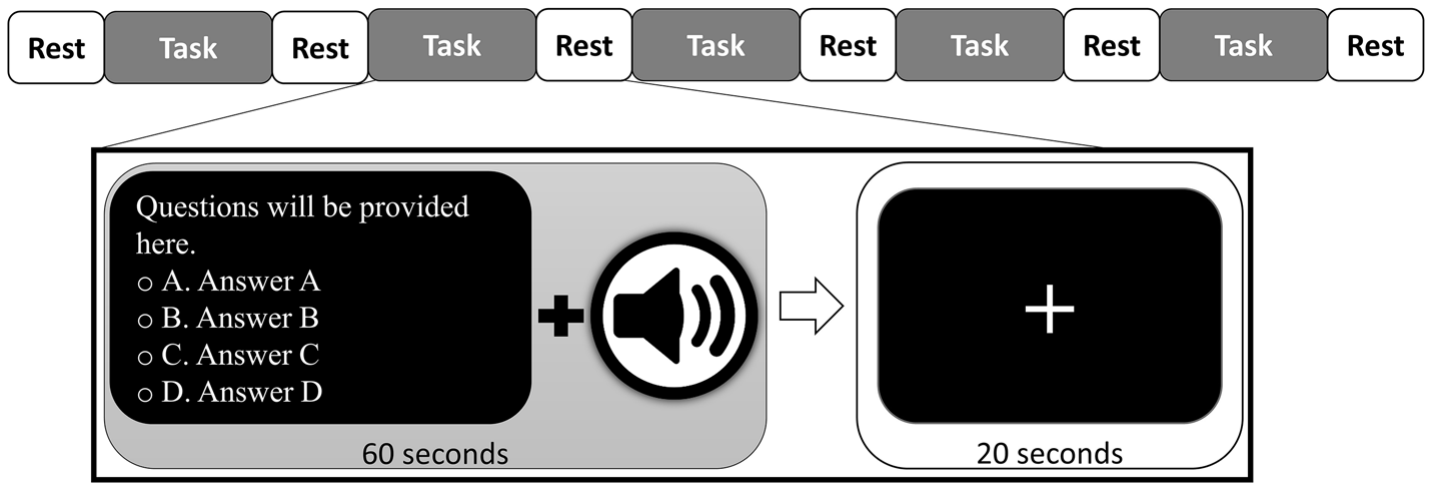
Task paradigm constructed for validation experiment.

This experiment consisted of two sessions differentiated by two sets of auditory emotional stimuli. All subjects performed the second experimental session at least six weeks after the first session. They repeated the experiment with another auditory emotional stimulus set. The order of the sessions was counterbalanced across the subjects. In each session, subjects sat approximately 60 cm in front of a monitor in a quiet, dimly lit room. As shown in Fig. 8, the experiment started with 20 s of rest followed by five alternate periods of task and rest. The subjects were required to focus on the on-screen cross and relax. During each 60 s task period, up to five questions about the nursing case study with four choices were displayed in succession on the monitor. Subjects were instructed to answer swiftly and complete as many questions as possible within a task period of 60 s. At the same time, auditory emotional stimuli were played through a speaker during the task periods. The types of the questions were retrieved based on five objectives proposed in Bloom’s taxonomy, including remembering, understanding, applying, analyzing, and evaluating^55^. The questions asked in both sessions were standardized in terms of type and difficulty level. The number of correctly solved questions, accuracy (percentage of correct answers over the total number of attempts), and response time were recorded and included in the statistical analysis.

### HRV analysis

The HRV is described as the fluctuation of distance between two successive heart beats (also known as normal-to-normal (NN) interval)^21,56^. HRV has been widely used as a quantitative marker to investigate the human autonomic nervous system (ANS) responses. Functioning as a physiological indicator of emotion processing^57,58^, it provides a non-invasive means to determine the balance between sympathetic (fight or flight) and parasympathetic (rest and digest) activity. The standard HRV analysis can be derived in both time and frequency domains^21,59^.

In this study, we focused on the time-domain HRV analysis as it demonstrated a better accuracy for short-term HRV recording^60^. We computed the *RMSSD* between normal heartbeats. Low *RMSSD* has been found to be associated with low parasympathetic activity due to poor emotional regulation^56,59^. We conducted HRV analysis using MATLAB-based (MathWorks Inc., Natick, MA) HRVTool v1.04 (https://github.com/MarcusVollmer/HRV)^61^. Firstly, as depicted in Fig. 3, the PPG signals were smoothened using moving average filter with window length equivalent to the sampling rate i.e. 200 data points. By setting the maximum and minimum heart rate to 180 and 60 beats per minute, respectively, the NN intervals were extracted from the preprocessed signals by using the QRS detection algorithm^62^. The artifacts (abnormal NN interval) were then eliminated using the same filtering method applied by Vollmer^61^. Ultimately, from the filtered NN intervals, we computed the HRV *RMSSD* by using the following formula:

9 $$RMSSD= \sqrt{\frac{1}{n-1}\sum_{i=1}^{n-1}{({NN}_{i+1}-{NN}_{i})}^{2}}$$

where *NN*_*i*_ denotes the time intervals of successive beats and *n* denotes the total number of normal peaks.

### Statistical analysis

Statistical analysis was conducted on the subjects’ behavioral performance, HRV and FC data. All multiple comparisons were FDR-adjusted using Benjamini and Hochberg method^63^ at desired *q*-level (FDR-adjusted *p* = 0.05).

#### Behavioral performance

We applied two-way mixed ANOVA to evaluate the group and emotional state effects on the behavioral performance indices (the number of correctly solved questions, accuracy and response time).

#### HRV

To examine the statistical differences in the *RMSSD*, we performed two-way mixed ANOVA to evaluate the interaction between emotional state and group type.

#### Comparison of functional connectivity methods

We compared the proposed semi-metric analysis to the weighted graph theory analysis in discriminating the emotional effect based on (1) ANOVA (2) correlation analysis.

In the entire PFC analysis, using IBM SPSS Statistics v23 (IBM Corp, Armonk, NY), two-way mixed ANOVA was conducted on the FC indices (*SMP*, *E*_*global*_, *E*_*local*_, *CC*, and *λ*) to examine the group and emotional task as the between-, and within-subjects factor respectively. At the regional level of semi-metric analysis and weighted graph theory analysis, three-way mixed ANOVA was carried out to evaluate the interaction between factors of group type, emotional state, and brain region (asymmetry). The effect size was determined using the partial eta squared (${\eta }_{p}^{2}$) and Cohen’s *d* for ANOVAs and pairwise comparisons, respectively.

Furthermore, to examine the association between stimulated emotional states and FC indices, we computed the Pearson’s correlation, *r* between the average changes in the *RMSSD* and the average changes in the global *SMP*, *E*_*global*_, *E*_*local*_, *CC*, and *λ* in affective versus neutral emotional states.

## Conclusion

This study explored semi-metric in analyzing fNIRS-based functional connectivity. The semi-metric analysis characterized the weighted FC by considering the information sharing paths at the global and regional levels of FC. The experimental results revealed that the semi-metric analysis, as correlated to HRV, was able to detect that the nursing students were more susceptible to emotional change. Under the affective condition, the nursing students demonstrated significant change in semi-metricity, but not in the conventional graph theory analysis. The results suggest the semi-metric analysis as an FC analytical technique could be generalized and extended to fNIRS. Further investigation on the age effect will help better understand about the underlying causes of reduced emotional sensitivity among the registered nurses.

## Supplementary Information

Supplementary Information.
