# Supplementary material for: fNIRS-based functional connectivity estimation using semi-metric analysis to study decision making by nursing students and registered nurses
Source: Sci Rep. 2020 Dec 16;10:22041. doi: 10.1038/s41598-020-79053-z (PMC7745044; doi:10.1038/s41598-020-79053-z)
Supplement: Supplementary file 1 — Supplementary Information. [file 41598_2020_79053_MOESM1_ESM.docx]

Supplementary Information

fNIRS-based Functional Connectivity Estimation using Semi-Metric Analysis to Study Decision Making by Nursing Students and Registered Nurses

Jie Sheng Chong^1^, Yee Ling Chan^1^, Esther G. M. Ebenezer^2^, Hoi Yen Chen^3^, Masashi Kiguchi^4^, Cheng-Kai Lu^1^, and Tong Boon Tang^1^

^1^Centre for Intelligent Signal and Imaging Research, Institute of Health and Analytics, Universiti Teknologi PETRONAS, Bandar Seri Iskandar 32610, Malaysia

^2^Medicine Based Department, Royal College of Medicine Perak, Universiti Kuala Lumpur, Ipoh 30450, Malaysia

^3^Nursing Programme, Royal College of Medicine Perak, Universiti Kuala Lumpur, Ipoh 30450, Malaysia

^4^Research & Development Group, Hitachi Ltd., Tokyo, Japan

*Correspondence: Tong Boon Tang, Centre for Intelligent Signal and Imaging Research, Institute of Health and Analytics, Universiti Teknologi PETRONAS, Bandar Seri Iskandar 32610, Malaysia (Email: [tongboon.tang@utp.edu.my](mailto:tongboon.tang@utp.edu.my))

# **Supplementary table 1.** ANOVA results in global semi-metric analysis based on HbR

| **Parameter** | **Comparison** | **Factors** | ***F*-values** | ***p*-values** | $\boldsymbol{\eta}_{\boldsymbol{p}}^{\boldsymbol{2}}$ |
| --- | --- | --- | --- | --- | --- |
| ***SMP*** | Two-way | Group x Task | 5.063 | 0.031 | 0.123 |
|  | Group (Nurses) | Task | 0.013 | 0.911 | 0.001 |
|  | Group (Students) | Task | 15.212 | 0.001 | 3.922 |

# **Supplementary table 2.** ANOVA results in regional semi-metric analysis based on HbR

| **Parameters** | **Comparison** | ***F*-values** | ***p*-values** | $\boldsymbol{\eta}_{\boldsymbol{p}}^{\boldsymbol{2}}$ |
| --- | --- | --- | --- | --- |
| ***SMP*** | ***Three-way*** |  |  |  |
|  | Group x Task x Region | 58.278 | 0.007 | 0.187 |
|  | ***Two-way (Nurses)*** |  |  |  |
|  | Task x Region | 0.106 | 0.749 | 0.006 |
|  | Task | 2.350 | 0.143 | 0.115 |
|  | Region | 0.489 | 0.493 | 0.026 |
|  | ***Two-way (Students)*** |  |  |  |
|  | Task x Region | 20.889 | <0.001 | 0.537 |
|  | Task | 13.454 | 0.002 | 0.428 |
|  | Region | 9.740 | 0.006 | 0.351 |

# **Supplementary table 3.** ANOVA results in global graph theory analysis based on HbR

| **Parameter** | **Comparison** | **Factors** | ***F*-values** | ***p*-values** | $\boldsymbol{\eta}_{\boldsymbol{p}}^{\boldsymbol{2}}$ |
| --- | --- | --- | --- | --- | --- |
| ***E_global_*** | Two-way | Group x Task | 0.042 | 0.839 | 0.001 |
|  | Main Effect | Task | 2.125 | 0.154 | 0.056 |
|  | Main Effect | Group | 0.077 | 0.757 | 0.003 |
| ***E_local_*** | Two-way | Group x Task | 0.041 | 0.840 | .001 |
|  | Main Effect | Task | 2.068 | 0.159 | 0.054 |
|  | Main Effect | Group | 0.097 | 0.783 | 0.002 |
| ***CC*** | Two-way | Group x Task | 0.041 | 0.840 | 0.001 |
|  | Main Effect | Task | 2.070 | 0.159 | 0.054 |
|  | Main Effect | Group | 0.077 | 0.783 | 0.001 |
| **λ** | Two-way | Group x Task | 0.005 | 0.944 | < 0.001 |
|  | Main Effect | Task | 2.375 | 0.062 | 0.062 |
|  | Main Effect | Group | 0.033 | 0.857 | 0.001 |

# **Supplementary table 4.** ANOVA results in regional graph theory analysis based on HbR

| **Parameters** | **Comparison** | ***F*-values** | ***p*-values** | $\boldsymbol{\eta}_{\boldsymbol{p}}^{\boldsymbol{2}}$ |
| --- | --- | --- | --- | --- |
| ***E_nodal_*** | ***Three-way*** |  |  |  |
|  | Group x Task x Region | 0.167 | 0.685 | 0.005 |
|  | Group x Task | 0.081 | 0.777 | 0.002 |
|  | Group x Region | 1.111 | 0.299 | 0.030 |
|  | Task x Region | 0.020 | 0.890 | 0.001 |
|  | Task | 1.995 | 0.166 | 0.053 |
|  | Region | 1.193 | 0.282 | 0.032 |
| ***CC*** | ***Three-way*** |  |  |  |
|  | Group x Task x Region | 0.207 | 0.652 | 0.006 |
|  | Group x Task | 0.093 | 0.762 | 0.003 |
|  | Group x Region | 1.140 | 0.293 | 0.031 |
|  | Task x Region | 0.005 | 0.944 | < 0.001 |
|  | Task | 1.947 | 0.171 | 0.051 |
|  | Region | 1.180 | 0.285 | 0.032 |
| **λ** | ***Three-way*** |  |  |  |
|  | Group x Task x Region | 0.380 | 0.541 | 0.010 |
|  | Group x Task | 0.076 | 0.785 | 0.002 |
|  | Group x Region | 1.319 | 0.258 | 0.035 |
|  | Task x Region | 0.022 | 0.882 | 0.001 |
|  | Task | 2.124 | 0.154 | 0.056 |
|  | Region | 1.115 | 0.298 | 0.030 |

# **Supplementary table 5.** Multiple-comparison results with FDR correction in global FC analyses based on HbR. The results are displayed in (FDR adjusted *p*-values; *t*-values; Cohen’s *d*). *df* represents degrees of freedom.

|  | **Comparisons** | ***df*** | ***SMP*** | ***Eglobal*** | ***Elocal*** | ***CC*** | ***λ*** |
| --- | --- | --- | --- | --- | --- | --- | --- |
| Students | Affective vs Neutral | 18 | 0.004; 3.922; 0.895* | 0.760; 0.900; 0.206 | 0.766; 0.894; 0.205 | 0.564; 0.895; 0.205 | 0.590; 1.079; 0.250 |
| Nurses | Affective vs Neutral | 18 | 0.911; 0.113; 0.026 | 0.760; 1.158; 0.275 | 0.766; 1.135; 0.275 | 0.564; 1.136; 0.250 | 0.590; 1.102; 0.252 |
| Affective | Nurses vs Students | 36 | 0.494; 1.178; 0.393 | 0.874; 0.159; 0.053 | 0.899; 0.129; 0.043 | 0.898; 0.129; 0.043 | 0.912;0.112; 0.037 |
| Neutral | Nurses vs Students | 36 | 0.548; -0.831; 0.277 | 0.847; 0.385; 0.128 | 0.899; 0.356; 0.119 | 0.898; 0.356; 0.119 | 0.912; 0.204; 0.068 |

# **Supplementary table 6.** Multiple-comparison results with FDR correction in regional FC analyses based on HbR. The results are displayed in (FDR adjusted *p*-values; *t*-values; Cohen’s *d*). *df* represents degrees of freedom.

| **Comparisons** | | | ***df*** | ***SMP*** | ***E_nodal_*** | ***CC*** | $\boldsymbol{\lambda}$ |
| --- | --- | --- | --- | --- | --- | --- | --- |
| Nurses | Affective | Right vs Left PFC | 18 | 0.604; 0.923; 0.212 | 0.907; 0.316; 0.066 | 0.928; 0.293; 0.057 | 0.961; 0.196; 0.033 |
|  | Neutral | Right vs Left PFC | 18 | 0.555; 0.919; 0.316 | 0.907; -0.326; 0.076 | 0.928; -0.332; 0.076 | 0.948; -0.382; 0.076 |
| Students | Affective | Right vs Left PFC | 18 | 0.036; 3.113; 0.716* | 0.876; 1.069; 0.258 | 0.858; 1.029; 0.229 | 0.814; 0.875; 0.204 |
|  | Neutral | Right vs Left PFC | 18 | 0.605; 1.036; 0.238 | 0.876; 0.949; 0.229 | 0.858; 0.982; 0.367 | 0.814; 1.114; 0.268 |
| Nurses | Right PFC | Affective vs Neutral | 18 | 0.806; 0.430; 0.099 | 0.876; 1.565; 0.344 | 0.858; 1.544; 0.194 | 0.814; 1.516; 0.344 |
|  | Left PFC | Affective vs Neutral | 18 | 0.605; 0.847; 0.194 | 0.876; 0.868; 0.194 | 0.858; 0.868; 0.115 | 0.814; 0.902; 0.212 |
| Students | Right PFC | Affective vs Neutral | 18 | 0.024; 3.298; 0.841* | 0.907; 0.555; 0.153 | 0.928; 0.459; 0.176 | 0.948; 0.466; 0.098 |
|  | Left PFC | Affective vs Neutral | 18 | 0.928; 0.125; 0.029 | 0.876; 0.793; 0.191 | 0.858; 0.809; 0.151 | 0.814; 0.997; 0.229 |
| Affective | Right PFC | Nurses vs Students | 36 | 0.928; -0.092; 0.031 | 0.907; -0.473; 0.158 | 0.928; -0.440; 0.147 | 0.948; -0.374; 0.125 |
|  | Left PFC | Nurses vs Students | 36 | 0.605; 0.765; 0.255 | 0.995; 0.007; 0.002 | 0.982; 0.022; 0.007 | 0.961; 0.050; 0.017 |
| Neutral | Right PFC | Nurses vs Students | 36 | 0.400; 1.734; 0.578 | 0.876; -0.798; 0.266 | 0.858; -0.819; 0.273 | 0.814; -0.840; 0.280 |
|  | Left PFC | Nurses vs Students | 36 | 0.605; 0.845; 0.232 | 0.876; -0.085; 0.028 | 0.982; -0.054; 0.018 | 0.961; 0.132; 0.044 |
